# Efficacy and safety of double endoscopy combined with exploration in the treatment of elderly patients with cholecystolithiasis complicated with choledocholithiasis

**DOI:** 10.1186/s12893-024-02352-z

**Published:** 2024-02-20

**Authors:** Jin Zhao, Xin Liu, Tao Huang

**Affiliations:** Department of General Surgery, Daxing Hospital of Capital Medical University, No.26, West Street, Huangcun Town, Daxing District, Beijing, 102600 China

**Keywords:** Laparoscopy, Choledochoscopy, Primary suture, Cholecystolithiasis, Choledocholithiasis

## Abstract

**Objective:**

To investigate the efficacy and safety of laparoscopy combined with choledochoscopy in the treatment of elderly patients with cholecystolithiasis complicated with choledocholithiasis.

**Methods:**

A retrospective analysis of 114 patients admitted to our hospital from January 2020 to January 2023 was conducted. These patients underwent laparoscopic cholecystectomy combined with choledocholithiasis and were divided into an elderly group (≥ 60 years old) of 63 cases and a young and middle-aged group (< 60 years old) of 51 cases according to age. The efficacy and safety indicators of the two groups of patients were observed, and complications were followed up by telephone within 6 months after surgery. The follow-up deadline was June 2023.

**Results:**

Among the eligible patients (53 men, 61 women, average age 57 years), all were successfully operated, and 1 case was converted to laparotomy. The elderly and young and middle-aged groups were compared concerning hospitalisation time, bowel sound recovery time, and total postoperative complications, and the differences were statistically significant (*P*-values were 0.009, 0.006, and 0.039). However, there was no statistically significant difference between the two groups of patients in terms of hospitalisation costs, intraoperative blood loss, operation time, drainage tube removal time, conversion to laparotomy rate, and stone clearance rate (*P*-values > 0 0.05).

**Conclusion:**

Strict adherence to surgical standards and enhanced postoperative care resulted in similar efficacy and safety results for double endoscopy combined with the exploration of treatment for elderly and young patients with cholecystolithiasis and choledocholithiasis.

## Introduction

The number of elderly patients with cholecystolithiasis and choledocholithiasis is increasing with the ageing of the population in China, presenting clinical challenges. These patients are typically treated with endoscopic retrograde cholangiopancreatography (ERCP) and subsequent elective laparoscopic cholecystectomy (LC) to prevent stones from entering the common bile duct (CBD) [1]. However, complications, such as residual stones and infections after LC, frequently occur.

With the technical development of laparoscopy and the increased importance attached to the preservation of the sphincter of Oddi (SO), LC combined with laparoscopic CBD exploration (LCBDE) is gradually being accepted as a treatment method, since both cholecystolithiasis and choledocholithiasis can be treated in one operation to avoid the risk of trauma and infections after multiple operations; furthermore, the SO is also saved from damage. However, postoperative complications increase after LC + LCBDE in elderly patients with additional underlying diseases, and postoperative recovery is also delayed in these patients due to their physical weakness. Studies [2] have shown that this risk can be avoided in young and middle-aged patients. Therefore, it is urgent to evaluate the safety and efficacy of LC + LCBDE in the treatment of diverse populations.

With the continuous development of minimally invasive surgery techniques, the treatment options for cholecystolithiasis with choledocholithiasis are constantly changing. The currently known methods include ERCP + LC and LC + LCBDE [3–7]. However, ERCP is associated with postoperative complications due to a damaged SO [8, 9] and increased difficulties in selective LC. Accordingly, LCBDE combined with LC can enable surgeons to address the two conditions at the same time while patients are anaesthetised only once, maximising the interests of patients with a higher success rate and lower postoperative recurrence. According to the latest treatment guidelines developed by the British Association of Upper Gastrointestinal Surgeons in 2017, LC + LCBDE has advantages such as short hospitalisation time and low cost and is recommended as the preferred treatment for this disease [10]. Therefore, LC + LCBDE has been recognised as an alternative to ERCP + LC in the treatment of cholelithiasis, with favourable efficacy and safety. However, the practicality of this method is still limited in elderly patients, and some clinicians suggest that LC + LCBDE should only be prioritised over ERCP + LC in young patients [11]. The tolerance to surgical trauma and anaesthesia is poor and postoperative recovery is slow in elderly patients due to the declined function of multiple organs. In some cases, LC + LCBDE has been successfully conducted in elderly patients by experienced surgeons [11–15]. However, the clinical efficacy and safety of LCBDE in elderly patients are less frequently evaluated compared with young patients, and the feasibility of this surgical procedure in elderly patients with cholecystolithiasis with choledocholithiasis has not been comprehensively evaluated. Accordingly, the present study was designed to investigate this issue.

## Materials and methods

### Study participants

The clinical and follow-up data of 114 patients with cholecystolithiasis and choledocholithiasis admitted to our department were retrospectively analysed. Based on their age, the patients were divided into the elderly group (≥ 60 years old) (*n* = 63) and the young and middle-aged group (< 60 years old) (*n* = 51).

The inclusion criteria comprised patients (1) who had been diagnosed with cholecystolithiasis and choledocholithiasis by B-mode ultrasonography or magnetic resonance imaging [16]; (2) undergoing selective laparoscopy combined with choledochoscopy; (3) aged ≥ 16 years; and (4) patients with no clinical manifestations of acute cholangitis; (5) preoperative ASA score level I or II.

The exclusion criteria included patients (1) complicated with intrahepatic stone; (2) with a previous history of biliary tract surgery; and (3) with severe preoperative cholangitis or biliary pancreatitis.

### Surgical procedure

**Fig. 1:**
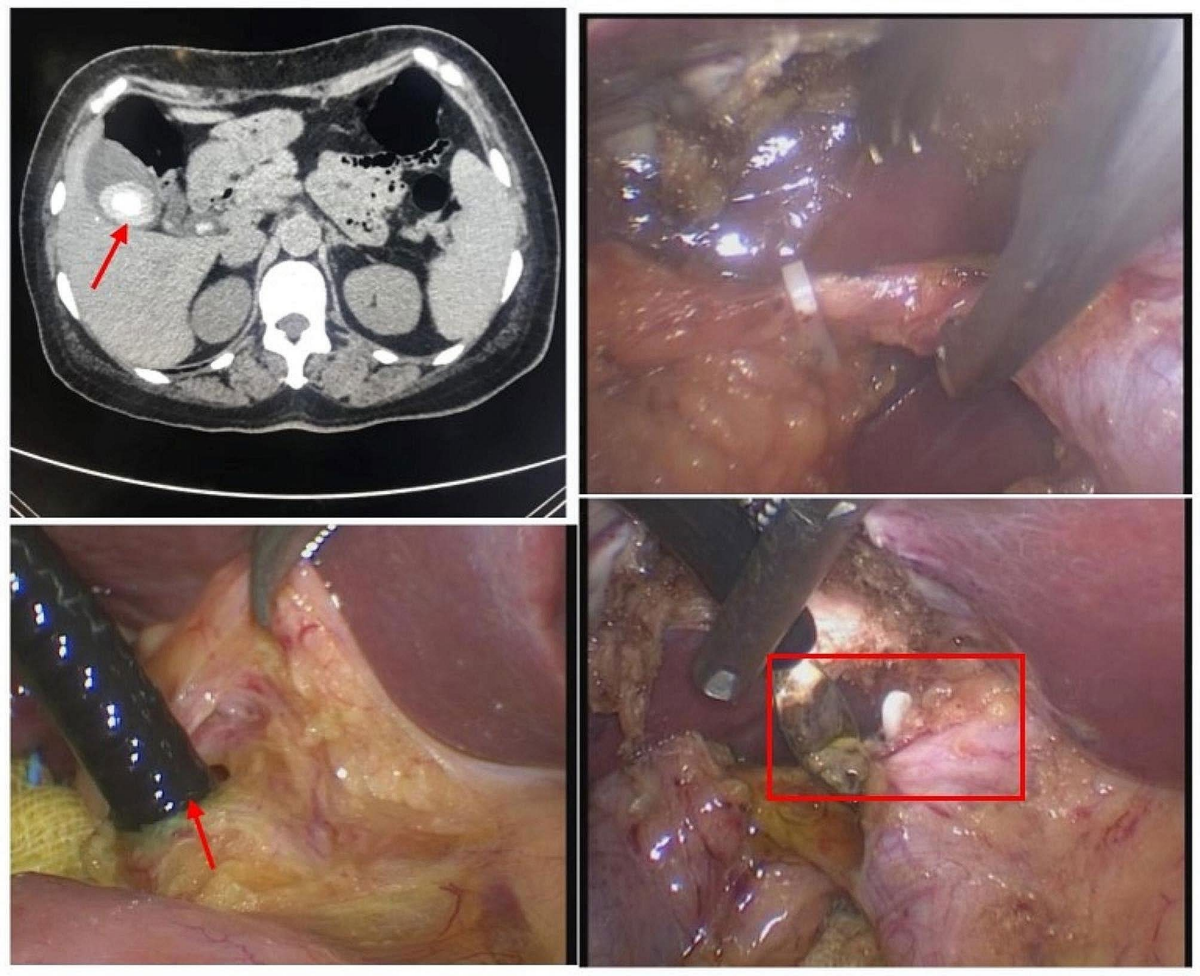
The surgical procedure of LC plus LCBDE. **A**: preoperative imaging suggesting cholecystolithiasis complicated with choledocholithiasis; **B**: LC; **C**: LCBDE after LC; and **D**: choledochoscopic removal of common bile duct stones

Relevant examinations and tests were completed for both patient groups after admission, and preoperative conventional symptomatic and supportive treatments such as infusion were given. The LC + LCBDE procedure was performed after surgical contraindications were excluded. Briefly, after general anaesthesia, patients were conventionally disinfected and draped, and a four-port method was used to establish pneumoperitoneum. The Calot triangle was dissected, and the gallbladder was lifted from the gallbladder ampulla. The bile duct and cystic artery were carefully separated, clamped and disconnected; the gallbladder was then removed via anterograde cholecystectomy. An electric needle-knife was used under a laparoscope to cut open the CBD, and a red urinary catheter was inserted to flush out the stones using pressure and normal saline. Then, choledochoscopy was used to explore the CBD up to the intrahepatic bile duct. The choledochoscopic examination was repeated to confirm that there was no obvious inflammation or CBD stenosis, followed by primary closure of the CBD. The CBD was closed by full-thickness suture using absorbable 4 − 0 Vicryl sutures, with a distance between stitches of 2–3 mm and 2 mm from the incision edge. Continuous suture was performed with double ligation using a knotter, and the CBD was checked for any leakage at the sutures [17]. Finally, after no active bleeding and biliary leakage were detected, an abdominal drainage tube was inserted via the epiploic foramen. The gallbladder and stones were removed through an incision under the xiphoid process. The abdominal drainage tube was removed based on postoperative conditions (Fig. 1).

### Outcome measures

The preoperative general data (sex, white blood cell, total bilirubin, albumin, ALT, AST, GGT, ALP, preoperative clinical manifestations, preoperative complications, number of stones in the gallbladder and CBD, CBD diameter) and surgical outcomes (surgical time, intraoperative blood loss, rate of conversion to laparotomy), as well as the postoperative general conditions (total hospitalisation cost, total length of stay, time of bowel sound recovery, time of drain removal, stone removal rate) and postoperative complications were recorded and analysed in detail. Postoperative complications were followed up by telephone for 6 months after the operation.

### Statistical analysis

The SPSS 22.0 software was used to conduct statistical analysis. Measurement data were presented as mean ± standard deviation, and an independent samples t-test was used for comparison between the two groups. Enumeration data were presented as percentages, the X^2^ test was used for comparison between the two groups, and Fisher’s exact test was used if necessary. Differences with a *P*-value < 0.05 were considered statistically significant.

## Results

### General data

A total of 114 eligible patients were selected for this study, including 53 men and 61 women, with a mean age of 57 years (range: 16–89 years). The operation was successfully completed for all patients, with conversion to laparotomy in 1 patient due to severe abdominal adhesions caused by a history of abdominal surgery. Of the 114 patients, 63 were included in the elderly group and 51 in the young and middle-aged group. The main clinical manifestations were epigastric pain, nausea and vomiting, fever, and jaundice; 23 patients were complicated with preoperative hypertension, 11 with diabetes, 5 with hyperlipidaemia, 2 with lung diseases, 4 with brain diseases, 7 with abdominal surgery history, and 2 with pancreatitis. The two groups were comparable, with no significant differences in preoperative general data (*P* > 0.05) (Table 1).

**Table 1:** Comparison of preoperative general data between the two groups

Variables	the elderly group (*n* = 63)	the young and middle-aged group(*n* = 51)	χ²/t	*P* value
Sex (M/F)	31/32	22/29	0.417	0.518
ALT(U/L)	119.2 ± 97.8	145.4 ± 101.6	-1.401	0.164
AST(U/L)	142.5 ± 203.8	141.9 ± 131.3	0.018	0.986
ALP(U/L)	358.0 ± 309.5	312.0 ± 199.1	0.958	0.340
Albumin(g/L)	35.3 ± 5.0	36.1 ± 5.3	-0.835	0.405
WBC (10^9^/l)	7.6 ± 5.3	7.7 ± 4.9	-0.044	0.965
TBIL(µmol/L)	50. 1 ± 51.6	51.8 ± 28.6	-0.223	0.824
GGT(U/L)	494.6 ± 530.5	529.4 ± 442.4	-0.375	0.708
Diameter of CBD (mm)	14.2 ± 3.6	13.5 ± 3.4	1.154	0.251
Choledocholithiasis (single/multiple)	23/40	17/34	0.125	0.844
Cholecystolithiasis (single/multiple)	35/28	29/22	0.020	1.0
Diabetes	8(12.7%)	3(5.9%)	1.502	0.340
Hypertension	16(25.4%)	7(13.7%)	2.384	0.160
Hyperlipidemia	1(1.6%)	4(7.8%)	2.630	0.171
Lung diseases	2(3.2%)	0(0%)	1.648	0.501
Brain diseases	3(4.8%)	1(2.0%)	0.653	0.627
History of abdominal surgery	4(6.3%)	3(5.9%)	0.011	1.0
Pancreatitis	1(1.6%)	1(2.0%)	0.023	1.0
Epigastric pain	60(95.2%)	46(90.2%)	1.098	0.464
Nausea and vomiting	21(33.3%)	13(25.5%)	0.828	0.414
Fever	4(6.3%)	6(11.8%)	1.033	0.339
Jaundice	7(11. 1%)	11(21.6%)	2.318	0.196

Note: patients in the elderly group were ≥ 60 years old and those in the young and middle-aged group were < 60 years old. ALT: Alanine aminotransferase; AST: Aspartate aminotransferase; ALP: Alkaline phosphatase; Tbil: Total bilirubin; GGT: Gamma-glutamyl transferase

### Surgical outcomes

There were no statistically significant differences in intraoperative blood loss, surgical time, and the rate of conversion to laparotomy between the two groups (*P* > 0.05) (Table 2).

**Table 2:** Comparison of surgical outcomes between the two groups

Variables	The elderly group(*n* = 63)	The young and middle -aged group(*n* = 51)	χ²/t	*P* value
Surgical time(min)	153.9 ± 74.4	162.5 ± 59.6	-0.668	0.506
Intraoperative blood loss (ml)	43.3 ± 50.6	38.4 ± 38.6	0.561	0.576
Conversion to laparotomy (%)	1(1.6%)	0(0%)	-	1.0

Note: patients in the elderly group were ≥ 60 years old and those in the young and middle-aged group were < 60 years old

### Postoperative general conditions

The total hospitalisation time and the time of bowel sound recovery were 18.7 ± 5.6 and 1.5 ± 0.8 days, respectively, in the elderly group, and 16.1 ± 4.7 and 1.2 ± 0.5 days, respectively, in the young and middle-aged group, and the differences between the two groups were statistically significant (*P* < 0.05). However, no significant differences in the total hospitalisation cost, time of drain removal, and stone removal rate were observed between the two groups (*P* > 0.05). Stone removal rate, an important index for the postoperative efficacy of LC + LCBDE, was observed between the two groups (*P* > 0.05) in the present study. There were 2 patients with residual stones in the elderly group (stone removal rate, 96.8%) and 1 patient with residual stones in the young and middle-aged group (stone removal rate, 98%), indicating that the postoperative efficacy was essentially the same for the two patient groups (Table 3).

**Table 3:** Comparison of postoperative general conditions between the two groups

Variables	The elderly group(*n* = 63)	The young and middle -aged group(*n* = 51)	χ²/t	*P* value
Total hospitalization cost (CNY)	40741.4 ± 14862.1	35844.6 ± 14231.1	1.783	0.077
Total length of stay(days)	18.7 ± 5.6	16. 1 ± 4.7	2.676	**0.009**
Time of bowel sound recovery(days)	1.5 ± 0.8	1.2 ± 0.5	2.788	**0.006**
Time of drain removal (days)	6.5 ± 3.0	5.5 ± 2.2	1.953	0.053
Stone removal rate (%)	61(96.8%)	50(98%)	0.162	1.0

Note: patients in the elderly group were ≥ 60 years old and those in the young and middle-aged group were < 60 years old. *P*-value in bold indicated a significant difference (*P* < 0.05)

### Postoperative complications

The overall incidence of complications in the elderly group was 15.9% (10/63). Four patients with postoperative pulmonary infection were given symptomatic anti-infection treatment during hospitalisation and discharged after recovery; 1 patient with postoperative abdominal bleeding was treated with an emergency laparotomy to stop bleeding, transferred to ICU for advanced life support treatment, and discharged after recovery; 2 patients with postoperative biliary leakage, and 1 patient each with postoperative intestinal ileus, biliary tract infection, and intestinal infection were given symptomatic supportive treatment and discharged after recovery. The overall incidence of complications in the young and middle-aged group was 3.9% (2/51). One patient with postoperative pulmonary infection was given symptomatic supportive treatment and discharged after recovery, and 1 patient with postoperative abdominal bleeding was treated with emergency laparotomy to stop bleeding, transferred to ICU, and discharged after recovery. There was a significant difference in the overall incidence of postoperative complications between the two groups (*P* < 0.05). All patients were followed up for 6 months after surgery; no complications were reported for any of the patients, except for 1 patient, who was lost to follow-up due to loss of contact (Table 4).

**Table 4:** Comparison of postoperative complications between the two groups

Variables	The elderly group (*n* = 63)	The young and middle-aged group (*n* = 51)	X^2^	*P* value
Pulmonary infection	4(6.3%)	1(2.0%)		
Abdominal bleeding	1(1.6%)	1(2.0%)		
Postoperative ileus	1(1.6%)	0(0%)		
Intestinal infection	1(1.6%)	0(0%)		
Biliary tract infection	1(1.6%)	0(0%)		
Biliary leakage	2(3.2%)	0(0%)		
Overall incidence	10(15.9%)	2(3.9%)	4.274	**0.039**

Note: patients in the elderly group were ≥ 60 years old and those in the young and middle-aged group were < 60 years old. *P*-value in bold indicated a significant difference (*P* < 0.05)

## Discussion

The clinical and follow-up data of 114 patients with cholecystolithiasis and choledocholithiasis who underwent LC + LCBDE with primary closure in the Department of Hepatobiliary and Pancreatic Surgery of our hospital between January 2020 and January 2023 were retrospectively analysed in this study to investigate the efficacy and safety of this treatment in elderly and young and middle-aged patients with these diseases. The results of the present study showed no statistically significant differences (*P* > 0.05) between the elderly and the young and middle-aged groups in terms of surgical time and intraoperative blood loss, indicating that the level of damage and bleeding and increased intraoperative surgical difficulties caused by LC + LCBDE did not change with age. On the contrary, the degree of damage was essentially the same. Moreover, conversion occurred in only 1 patient in the elderly group, mainly due to a history of upper abdominal surgery and severe abdominal adhesions, which increased the difficulty of the surgery and resulted in the conversion to laparotomy. These results suggested that this risk can be completely avoided if the patients are reasonably selected.

In the present study, the total length of stay and the time of bowel sound recovery in the elderly group were slightly increased compared with the young and middle-aged group; however, no statistical significances were observed in the total hospitalisation cost and the time of drain removal. The relevant reasons mainly included the physical weakness of these elderly patients, the slow postoperative recovery of gastrointestinal peristalsis, and delayed overall rehabilitation progress. In addition, caution was needed in allowing the discharge of these patients from the hospital, and the discharge time was generally intervened. Preoperative comprehensive evaluation programmes were required for each patient, especially elderly patients. The surgery was only performed in elderly patients if the results of all examinations were acceptable, which led to a delayed hospital stay. Similarly, caution was needed for increased the time of drain removal and the total hospitalisation cost of the two groups. However, the present study showed that this difference was controllable. If the preoperative examination time was strictly controlled in these elderly patients, the discharge time, as well as the time of drain removal among these patients were essentially the same as those of the young and middle-aged group, with a reduced total hospitalisation cost.

Studies have shown that a high stone removal rate can be attributed to the use of choledochoscopy, electrohydraulic lithotripsy (EHL), and a balloon dilation system [15]. Although intraoperative ultrasound and cholangiography are helpful in the detection of common bile duct stones [18, 19], EHL and laser lithotripsy can significantly improve the stone removal rate. However, residual stones inevitably present in a small number of patients. In the present study, a few patients in both groups had residual stones, which were caused by incomplete EHL, and this finding was consistent with previous studies [20, 21]. All of the patients with residual stones were identified in postoperative follow-up and eventually recovered after they returned for a second operation.

In the present study, the overall incidence of postoperative complications was 15.9% in the elderly group and 3.9% in the young and middle-aged group, including surgical site infections, bleeding, bile duct injuries, and respiratory issues, and the difference was statistically significant. Zhang et al. [22] reported an overall incidence of complications of 18.5% in elderly patients undergoing LC + LCBDE, and Zhou et al. [8]reported an incidence of 14.8%, which was consistent with the findings of the present study. In the present study, pulmonary infection was the major and most common complication in these elderly patients. This was explained by the more severe conditions and a higher probability of infection in elderly patients. Moreover, the preoperative prophylactic use of antibiotics and adjunctive drugs to improve respiratory function was observed to reduce the incidence of postoperative pulmonary complications to some extent, and an incidence of pulmonary complications as low as that of young and middle-aged patients could be achieved. Additionally, 2 patients with postoperative biliary leakage in the elderly group were given symptomatic treatment and discharged after recovery. Biliary leakage, a very rare and serious complication requiring an emergency laparotomy, had possibly been caused in these patients by the thin wall of the CBD and the use of primary closure after incision of the CBD. However, according to relevant studies, biliary leakage is controllable to some degree and can be prevented by meticulous surgical skills and effective postoperative care [23]. Moreover, based on the findings concerning biliary leakage in the present study, it is recommended that an incision at the junction of the CBD, cystic duct, and common hepatic duct (where blood vessels are less densely distributed) can further reduce the damage of electrocoagulation to the bile duct at the bleeding point and, subsequently, the incidence of biliary leakage. In addition, studies have found that the diameter of the CBD and insufficient surgical experience, rather than the age of patients, are risk factors for postoperative biliary leakage [24], which is consistent with the findings of the present study. These results suggest that advanced age alone should not be a contraindication for LC + LCBDE surgery. Furthermore, studies [25–30] also reported a special intraoperative technique as follows: after the continuous or intermittent suture of the incision site on the CBD was completed, intermittent suture should routinely be added to the surface of this site. This may help reduce tension, which could serve as a reason for reduced biliary leakage [31, 32]. The reliability of this technique requires further verification in future studies. One patient in each group in the current study experienced postoperative abdominal bleeding. Both patients were treated with an emergency laparotomy to stop the bleeding, transferred to the ICU for advanced life support, and subsequently discharged after recovery.

These results demonstrate that the same efficacy and safety can be achieved in elderly patients and young and middle-aged patients by strictly controlling surgical contraindications and strengthening intraoperative surgical procedures and postoperative care.

The current study has some limitations. The sample size was relatively small and may not represent the characteristics and conditions of the entire elderly patient population; as such, the reliability and generalisation of the results could be impaired. This study was conducted in one medical centre, and the practices and treatment programmes in this hospital may not reflect those of other medical institutions. Intervention measures and follow-up processes are not controllable in a retrospective design, and potential biases may exist. It was also impossible to evaluate the relative efficacy and safety of LC + LCBDE treatment because it was not compared with other treatment methods or an observation group. Therefore, future multicentre and randomised controlled studies with long-term follow-up periods and large samples are needed to evaluate the efficacy and safety of this method.

## Conclusion

This study preliminarily found that LC + LCBDE is feasible and effective in the treatment of cholelithiasis in elderly patients. The risk of complications can be reduced through reasonable preoperative evaluation and nursing intervention. Therefore, the safety of this treatment in elderly patients closely matches that in young and middle-aged patients.

## Data Availability

No datasets were generated or analysed during the current study.
